# High inter-rater reliability of Japanese bedriddenness ranks and cognitive function scores: a hospital-based prospective observational study

**DOI:** 10.1186/s12877-021-02108-x

**Published:** 2021-03-09

**Authors:** Masaki Tago, Naoko E. Katsuki, Shizuka Yaita, Eiji Nakatani, Shun Yamashita, Yoshimasa Oda, Shu-ichi Yamashita

**Affiliations:** 1 Department of General Medicine, Saga University Hospital, 5-1-1 Nabeshima, Saga, 849-8501 Japan; 2 Division of Statistical Analysis, Research Support Center, Shizuoka General Hospital, Shizuoka, Japan; 3 Translational Research Center for Medical Innovation, Foundation for Biomedical Research and Innovation at Kobe, Kobe, Japan; 4 Department of General Medicine, Yuai-Kai Foundation and Oda Hospital, Kashima, Japan

**Keywords:** Barthel index, Bedriddenness ranks, Cognitive function scores, Concordance rate, Cronbach’s α, Inter-rater reliability, Intraclass correlation coefficient, Kappa coefficient, Katz index, Spearman’s correlation test

## Abstract

**Background:**

The statistical validities of the official Japanese classifications of activities of daily living (ADLs), including bedriddenness ranks (BR) and cognitive function scores (CFS), have yet to be assessed. To this aim, we evaluated the ability of BR and CFS to assess ADLs using inter-rater reliability and criterion-related validity.

**Methods:**

New inpatients aged ≥75 years were enrolled in this hospital-based prospective observational study. BR and CFS were assessed once by an attending nurse, and then by a social worker/medical clerk. We evaluated inter-rater reliability between different professions by calculating the concordance rate, kappa coefficient, Cronbach’s α, and intraclass correlation coefficient. We also estimated the relationship of the Barthel Index and Katz Index with the BR and CFS using Spearman’s correlation coefficients.

**Results:**

For the 271 patients enrolled, BR at the first assessment revealed 66 normal, 10 of J1, 15 of J2, 18 of A1, 31 of A2, 37 of B1, 35 of B2, 22 of C1, and 32 of C2. The concordance rate between the two BR assessments was 68.6%, with a kappa coefficient of 0.61, Cronbach’s α of 0.91, and an intraclass correlation coefficient of 0.83, thus showing good inter-rater reliability. BR was negatively correlated with the Barthel Index (r = − 0.848, *p* < 0.001) and Katz Index (r = − 0.820, *p* < 0.001), showing justifiable criterion-related validity.

Meanwhile, CFS at the first assessment revealed 92 normal, 47 of 1, 19 of 2a, 30 of 2b, 60 of 3a, 8 of 3b, 8 of 4, and 0 of M. The concordance rate between the two CFS assessments was 70.1%, with a kappa coefficient of 0.62, Cronbach’s α of 0.87, and an intraclass correlation coefficient 0.78, thus also showing good inter-rater reliability. CFS was negatively correlated with the Barthel Index (r = − 0.667, *p* < 0.001) and Katz Index (r = − 0.661, *p* < 0.001), showing justifiable criterion-related validity.

**Conclusions:**

BR and CFS could be reliable and easy-to-use grading scales of ADLs in acute clinical practice or large-scale screening, with high inter-rater reliabilities among different professions and significant correlations with well-established, though complicated to use, instruments to assess ADLs.

**Trial registration:**

UMIN000041051 (2020/7/10).

**Supplementary Information:**

The online version contains supplementary material available at 10.1186/s12877-021-02108-x.

## Background

In the early 1990s, the Ministry of Health, Labour and Welfare (MHLW) released classifications for activities of daily living (ADLs), consisting of bedriddenness ranks and cognitive function scores [1]. These classifications have been widely used under the Japanese Health Insurance and Nursing-care Insurance systems, by attending physicians of elderly patients to assess them for nursing care insurance eligibility [2], by certified evaluators of long-term care to assess persistent disabilities of patients in their own home, or by attending nurses to determine the level of hospital care or discharge support required by elderly inpatients [3–5]. Bedriddenness ranks assess the degree of bedriddenness, which is classified into nine grades using four classification steps (S1, Fig. A) [5]. Cognitive function scores are used to evaluate cognitive impairment, which is classified into eight grades using five classification steps (S1, Fig. B) [5]. In aged societies with limited medical and nursing care resources, such as Japan, easily obtainable bedriddenness ranks and cognitive function scores could be used as comprehensive ADL indicators to screen at-risk older people for their daily living assistance requirements. Furthermore, bedriddenness ranks and cognitive function scores could be used as common tools between hospital or senior care home settings and home or local community settings of patients. While it would be ideal to gather information about specific basic ADLs of all older patients, many are left unassessed due to the overwhelmingly large number of requirements needed to even qualify for an assessment in Japan, which is globally at the forefront of our aged society. Other developed countries with an aged society will certainly experience the same situation as Japan in the near future. These classifications are already used to evaluate ADLs in older patients in common settings, such as hospitals, nursing-care facilities, and local communities, to provide patients with consecutive, long-term care in Japan.

Other than the MHLW bedriddenness ranks and cognitive function scores, several standard and established scores of ADLs and impairment of cognitive function MHLW have been reported. These include the Barthel Index (BI), which classifies the abilities of 10 basic ADLs into two to four grades [6], the Katz Index (KI), which classifies six basic ADLs into two grades (an independent or care-requiring condition), and the Mini-Mental State Examination (MMSE), which evaluates 11 items (e.g. naming objects, calculation, and manual dexterity while drawing). The BI, KI, and MMSE are well-established measures; however, they are complex and can be difficult to implement. Indeed, despite their high inter-rater reliability [7, 8], they are complicated and time-consuming to perform – particularly the BI and MMSE [9, 10] – which makes these measures difficult to use in an acute care hospital setting or to evaluate many subjects, such as mass screening [3–5, 11]. On the other hand, bedriddenness ranks and cognitive function scores are extremely simple and easy to implement, and could therefore be more suited to such situations [3–5, 11]. Despite their routine use in daily clinical practice and in research settings in Japan [3–5, 11], the precision and validation of bedriddenness ranks and cognitive function scores have yet to be established.

We herein report the usefulness of the MHLW bedriddenness ranks and cognitive function scores as tools to assess ADLs. For this, we examined inter-rater reliability and criterion-related validity in a prospective investigation of inpatients at an acute care hospital in a suburban city in Japan.

## Methods

### Study design and patients

This study was a hospital-based prospective observational study. The subjects were inpatients aged 75 years or older at Yuaikai Oda Hospital (S2, Appendix.), an acute care hospital in a suburban city in Japan, who were admitted from November 2017 to September 2019. The following patients were excluded: those who did not consent to participate in the study, those whose hospital stay was shorter than 24 h, those who were in a serious or possibly fatal condition, and/or those with miscellaneous conditions that made evaluation impossible. A serious or possibly fatal condition was defined as having a disturbance of consciousness equal to or more severe than III-100 of the Japan Coma Scale within 72 h of admission, meaning that they were unable to open their eyes or take action to avoid painful stimuli [12], had poor vital signs, with a shock index > 1 (i.e., pulse rate divided by systolic blood pressure > 1) [13], showed percutaneous oxygen saturation < 90%, and required administration of 8 L/min or more of oxygen.

### Data and data sources

The variables we checked from the medical records on admission were age, sex (male or female), ambulance transfer (presence or absence), admission with a referral letter from a primary physician (presence or absence), duration of hospitalization (days), department of admission (Internal Medicine, Surgery, Cardiology, Dermatology, Otorhinolaryngology, Neurosurgery), MHLW bedriddenness rank and cognitive function score, emergency admission (presence or absence), place of abode (home, nursing home, or other place), basic ADLs (eating, moving, personal maintenance, going to the toilet, bathing, walking, going up and down the stairs, dressing, defecation, and urination; independently or not), and diseases causing hospitalization (according to the International Classification of Diseases, 10th edition: ICD-10). Furthermore, we calculated the BI and KI using data on basic ADLs that were routinely and systematically assessed in each patient on admission by an attending nurse.

Bedriddenness ranks were classified into five grades, which were further divided into nine grades, as follows: normal, J (J1, J2), A (A1, A2), B (B1, B2), and C (C1, C2) (S1, Fig. A). Cognitive function scores were classified into six grades, which were further divided into eight grades, as follows: normal, 1, 2 (2a, 2b), 3 (3a, 3b), 4, and M (S1, Fig. B) [5, 14]. In this study, MHLW bedriddenness ranks and cognitive function scores were checked twice; at Oda hospital, bedriddenness ranks and cognitive function scores of all inpatients aged 75 years or older are routinely checked by an attending nurse within 24 h after admission, which are subsequently confirmed by another nurse in charge of discharge support care, with results recorded in the medical chart both times. The results of this routine check of bedriddenness ranks and cognitive function scores were derived from the medical charts for Assessment 1. For Assessment 2, these results were checked independently within 72 h after admission by a medical social worker or a medical clerk of the hospital, i.e. a person of a medical profession other than a nurse, who was blinded to the results of Assessment 1. The flowchart shown by in supplemental Figure 1 (S1, Fig.) shows the scoring procedure for both Assessment 1 and 2. Although the criterion of Bedriddenness rank J is “A person who has mild disorders, but who is almost independent in activities of daily living and can go out using public transport (J1) or around the neighborhood (J2)”, bedriddenness ranks were evaluated at the patients’ bedside in the hospital, for both Assessment 1 and 2. These evaluations were made according to information obtained during interviews with the patient or family members, or observations about the patient’s condition. For Assessment 1, a referral letter or nursing records were also used in these evaluations. All variables other than the results of Assessment 2 were collected from medical charts.

### Statistical analysis

The background characteristics of all the eligible patients were analyzed according to the grade of bedriddenness ranks or cognitive function scores. Categorical background characteristic variables are presented as the numbers of patients and percentages, and continuous background characteristic variables as the median and quartile ranges. Categorical variables were analyzed using the Chi-square test, and continuous variables were analyzed using an analysis of variance and F-test.

After excluding patients with missing data in Assessment 2, the concordance rate, kappa coefficient, Cronbach’s α, and intraclass correlation coefficient between Assessment 1 and 2 for both the bedriddenness ranks and cognitive function scores were calculated. The correlations of bedriddenness ranks/cognitive function scores with BI and KI were analyzed using Spearman’s rank correlation coefficient.

Statistical significance was set at *p* < 0.05. IBM SPSS Statistics (version 25, IBM, Armonk, New York, USA) was used for the statistical analyses.

### Ethical considerations

This study conforms to Ethical Guidelines for Medical and Health Research Involving Human Subjects issued by the Japanese MHLW and the Ministry of Education, Culture, Sports, Science, and Technology. This study was approved by the research ethics committee of the Yuai-kai Foundation and Oda Hospital (No. 20171201). We obtained consent from each patient individually, and anonymity of the patients was protected.

## Results

### Enrollment and background characteristics of patients

**Fig. 1:**
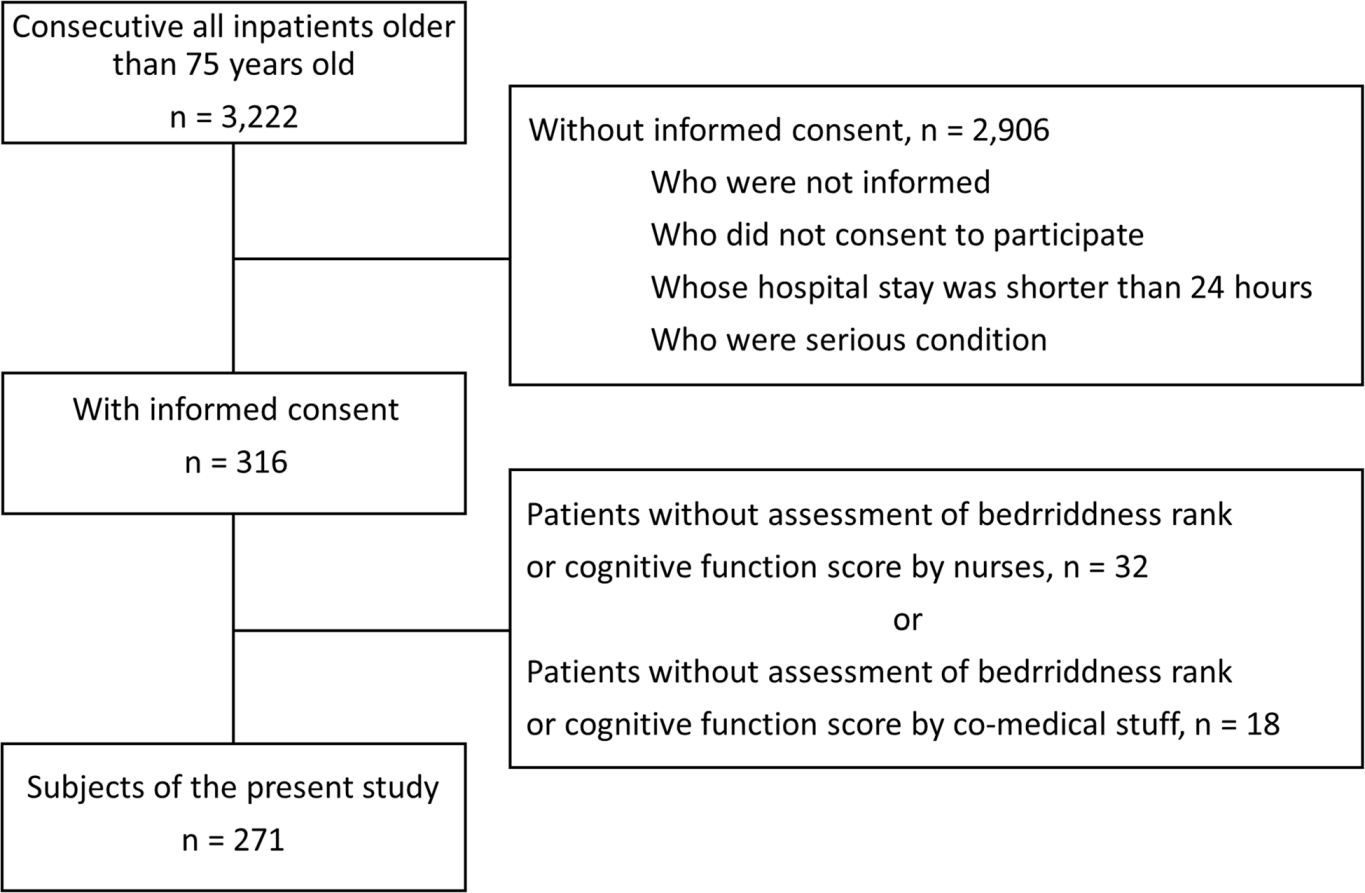
Data flow diagram

During the study period**,** 3222 inpatients aged 75 years or older were admitted, 2906 of whom were excluded according to the exclusion criteria. After excluding patients with missing data, 271 patients were eligible to be enrolled (Fig. 1). Table 1 shows the background characteristics of patients. The median age of patients was 86 years, 114 (42.1%) were men, and the majority of patients (174; 64.2%) were admitted to the department of Internal Medicine.

**Table 1:** Characteristics of enrolled patients

Variables	n	% (interquartile)
Age (year)	86	(81–90)
Gender, Male	114	42.1
Admitted by ambulance	37	13.7
Presence of referral medical letter	107	39.5
Length of hospital stay (day)	13	(8–22)
Department		
Internal medicine	174	64.2
Surgery	34	12.5
Cardiovascular surgery	33	12.2
Dermatology	14	5.2
Plastic surgery	6	2.2
Otolaryngology	5	1.8
Neurosurgery	5	1.8

Categorical variables are presented as the number of patients and percentages, and continuous variables as the median and quartile range

Assessment 1 revealed the following: bedriddenness rank frequencies: 66 normal, 10 J1, 15 J2, 18 A1, 31 A2, 37 B1, 35 B2, 22 C2, and 32 C2; cognitive function score frequencies: 92 normal, 47 1, 19 2a, 30 2b, 60 3a, 8 3b, 8 4, and 0 M. More detailed analyses of background characteristics according to bedriddenness ranks or cognitive function scores showed differences according to age, sex, emergency admission, pre- and post-hospitalization living locations, and duration of hospitalization (Tables 2 and 3). The diseases causing hospitalization consisted of 15 conditions among the major categories, and 144 diseases among the subcategories of the ICD-10. The condition with the highest incidence, 63 cases, among the major categories was cardiovascular diseases, while the disease with the highest incidence, 22 cases, among the subcategories was congestive heart failure.

**Table 2:** Characteristics according to bedriddenness rank

Bedriddenness rank (n)	Normal (66)	J1 (10)	J2 (15)	A1 (18)	A2 (31)	B1 (37)	B2 (35)	C1 (22)	C2 (32)	***p*** value^a^
Age (year)	82 (78–87)	85 (81–91)	88 (84–91)	82 (80–90)	87 (81–90)	88 (87–91)	89 (82–93)	88 (85–93)	87 (83–92)	< 0.001
Gender, Male	42 (64%)	4 (40%)	1 (7%)	10 (56%)	10 (32%)	12 (32%)	13 (37%)	11 (50%)	9 (28%)	0.001
Admitted by ambulance	2 (3%)	0 (0%)	1 (7%)	4 (22%)	7 (23%)	3 (8%)	6 (17%)	7 (32%)	6 (19%)	0.010
Presence of referral medical letter	15 (23%)	3 (30%)	6 (40%)	8 (44%)	11 (36%)	18 (49%)	14 (40%)	10 (46%)	19 (59%)	0.050
Department: Internal medicine	34 (52%)	8 (80%)	7 (47%)	11 (61%)	21 (67%)	20 (54%)	28 (80%)	15 (68%)	26 (81%)	0.182
General surgery	8 (12%)	1 (10%)	5 (33%)	4 (22%)	4 (13%)	6 (16%)	1 (3%)	2 (9%)	3 (9%)	–
Cardiovascular Surgery	14 (21%)	0 (0%)	1 (7%)	1 (6%)	3 (10%)	7 (19%)	2 (6%)	4 (18%)	1 (3%)	–
Place of abode before admission: Home	65 (99%)	9 (90%)	15 (100%)	14 (78%)	27 (87%)	26 (70%)	30 (86%)	18 (82%)	16 (50%)	< 0.001
Care facility	0 (0%)	1 (10%)	0 (0%)	3 (17%)	3 (10%)	5 (14%)	2 (6%)	1 (5%)	1 (3%)	–
Hospital	1 (2%)	0 (0%)	0 (0%)	1 (6%)	1 (3%)	6 (16%)	3 (9%)	3 (14%)	15 (47%)	–
Duration of hospitalization (day)	9 (5–12)	8 (5–22)	12 (3–18)	11 (9–17)	16 (9–25)	14 (8–22)	16 (11–22)	12 (7–23)	26 (12–40)	< 0.001
Place of abode after discharge: Home	53 (95%)	5 (63%)	13 (100%)	11 (65%)	21 (81%)	16 (50%)	16 (46%)	7 (37%)	2 (7%)	< 0.001
Care facility	1 (2%)	1 (13%)	0 (0%)	2 (12%)	3 (12%)	12 (38%)	13 (37%)	6 (32%)	7 (26%)	–
Hospital	1 (2%)	1 (13%)	0 (0%)	3 (18%)	1 (4%)	1 (3%)	4 (11%)	2 (11%)	10 (37%)	–
Death within 30 days after discharge	1 (2%)	1 (14%)	0 (0%)	0 (0%)	0 (0%)	2 (6%)	1 (3%)	2 (12%)	4 (17%)	0.059

Categorical variables are presented as the number of patients and percentages, and continuous variables as the median and quartile range

^a^For continuous and categorical variables, between-group comparisons were made using the F-test for analysis of variance and the Chi-square test, respectively

**Table 3:** Characteristics according to cognitive function score

Cognitive function score (n)	Normal (92)	1 (47)	2a (19)	2b (30)	3a (60)	3b (8)	4 (8)	M (0)	Unknown (7)	***p*** value^a^
Age (year, median)	82 (78–87)	88 (81–90)	90 (83–92)	88 (82–92)	89 (85–93)	88 (86–94)	84 (81–89)	–	82 (−)^b^	< 0.001
Gender, Male	47 (51%)	22 (47%)	5 (26%)	11 (37%)	19 (32%)	3 (38%)	4 (50%)	0 (0%)	3 (43%)	0.272
Admitted by ambulance	6 (7%)	10 (21%)	2 (11%)	3 (10%)	10 (17%)	2 (25%)	3 (38%)	0 (0%)	1 (14%)	0.101
Presence of referral medical letter	23 (25%)	22 (47%)	7 (37%)	11 (37%)	31 (52%)	5 (63%)	4 (50%)	0 (0%)	4 (57%)	0.024
Department: Internal medicine	50 (54%)	30 (64%)	15 (79%)	18 (60%)	48 (80%)	5 (63%)	3 (38%)	0 (0%)	5 (71%)	0.323
General surgery	15 (16%)	7 (15%)	2 (11%)	2 (7%)	4 (7%)	3 (38%)	1 (13%)	0 (0%)	0 (0%)	–
Cardiovascular Surgery	14 (15%)	7 (15%)	2 (11%)	5 (17%)	3 (5%)	0 (0%)	1 (13%)	0 (0%)	1 (14%)	–
Place of abode before admission: Home	85 (92%)	42 (89%)	16 (84%)	27 (90%)	38 (63%)	4 (50%)	6 (75%)	0 (0%)	4 (57%)	< 0.001
Care facility	5 (5%)	3 (6%)	1 (5%)	0 (0%)	4 (7%)	2 (25%)	1 (13%)	0 (0%)	0 (0%)	–
Hospital	2 (2%)	2 (4%)	2 (11%)	3 (10%)	18 (30%)	2 (25%)	1 (13%)	0 (0%)	3 (43%)	
Duration of hospitalization (day)	10 (6–17)	12 (7–23)	14 (11–27)	13 (8–20)	16 (10–26)	16 (11–23)	19 (4–39)	–	7 (−)^b^	0.010
Place of abode after discharge: Home	70 (89%)	27 (66%)	9 (50%)	14 (54%)	19 (35%)	2 (29%)	2 (33%)	0 (0%)	2 (29%)	< 0.001
Care facility	5 (6%)	5 (12%)	4 (22%)	8 (31%)	16 (30%)	4 (57%)	2 (33%)	0 (0%)	3 (43%)	–
Hospital	3 (4%)	5 (12%)	2 (11%)	1 (4%)	9 (17%)	1 (14%)	2 (33%)	0 (0%)	1 (14%)	–
Death within 30 days after discharge	1 (1%)	2 (5%)	1 (6%)	0 (0%)	7 (14%)	0 (0%)	0 (0%)	0 (0%)	1 (14%)	0.079

Categorical variables are presented as the number of patients and percentages, and continuous variables as the median and quartile range

^a^ For continuous and categorical variables, between-group comparisons were made using the F-test for analysis of variance and the Chi-square test, respectively

^b^ This category has no quartile range

### Inter-rater reliability of the MHLW bedriddenness ranks

The concordance rate of bedriddenness ranks between Assessment 1 and 2 was 68.6%, kappa coefficient 0.61, Cronbach’s α 0.91, and intraclass correlation coefficient 0.83 (95% confidence interval (CI): 0.79–0.86). The concordance rates of Assessment 1 and 2 in each grade of bedriddenness ranks in descending order were 87.5% for C2, 75.8% for Normal, 73.0% for B1, 71.0% for A2, 68.2% for C1, 65.7% for B2, and less than 50% for J1, J2, and A1 (Table 4). There was no significant difference in concordance rates of bedriddenness ranks between Assessment 1 and 2 among the major categories of the ICD-10 (Chi-square test, χ^2^ = 11.1, *p* = 0.681, S3, Table.).

**Table 4:** Differences of bedriddenness rank between assessments by nurses and by other staff

Bedriddenness rank			Assessment 2								Total		Concordance rate %
		Normal	J1	J2	A1	A2	B1	B2	C1	C2	n	%	
Assessment 1	Normal	50	8	1	2	4	0	1	0	0	66	24.4	75.8
	J1	2	3	1	1	2	0	0	0	1	10	3.7	30.0
	J2	3	1	6	4	0	0	1	0	0	15	5.5	40.0
	A1	3	2	1	7	2	1	1	0	1	18	6.6	38.9
	A2	2	1	3	2	22	0	1	0	0	31	11.4	71.0
	B1	3	0	0	1	3	27	2	1	0	37	13.7	73.0
	B2	2	1	0	0	5	4	23	0	0	35	12.9	65.7
	C1	1	1	0	0	2	1	2	15	0	22	8.1	68.2
	C2	0	0	0	0	1	1	2	0	28	32	11.8	87.5
	Unknown	1	0	0	0	0	0	1	0	3	5	1.8	NA
Total		67	17	12	17	41	34	34	16	33	271	100	68.6

The bedriddenness rank, routinely checked by a nurse, was derived from the medical charts for Assessment 1. In Assessment 2, bedriddenness was checked independently within 72 h after admission, and without knowledge of the Assessment 1 results, by a medical social worker or a medical clerk, i.e. a person of a medical profession other than a nurse

### Inter-rater reliability of the MHLW cognitive function scores

The concordance rate of cognitive function scores between Assessment 1 and 2 was 70.1%, kappa coefficient 0.62, Cronbach’s α 0.87, and intraclass correlation coefficient 0.78 (95% CI: 0.73–0.82). No patients were classified as having a cognitive function score M in Assessment 1. The concordance rates of Assessment 1 and 2 in each grade of cognitive function scores in descending order were 87.5% for 3b, 85.9% for Normal, 75% for 3a, 75% for 4, 63.8% for 1, and less than 50% for 2a and 2b (Table 5). There was no significant difference in concordance rates of cognitive function scores between Assessment 1 and 2 among the major categories of the ICD-10 (Chi-square test, χ^2^ = 14.5, *p* = 0.415, S3, Table.).

**Table 5:** Differences of cognitive function score between assessments by nurses and by other staff

Cognitive function score			Assessment 2								Total		Concordance rate %
		Normal	1	2a	2b	3a	3b	4	M	Unknown	n	%	
Assessment 1	Normal	79	7	3	0	1	1	0	0	1	92	33.9	85.9
	1	10	30	2	1	3	1	0	0	0	47	17.3	63.8
	2a	4	2	9	1	3	0	0	0	0	19	7.0	47.4
	2b	3	3	6	14	2	2	0	0	0	30	11.1	46.7
	3a	0	1	1	10	45	2	1	0	0	60	22.1	75.0
	3b	0	0	1	0	0	7	0	0	0	8	3.0	87.5
	4	0	0	0	0	0	1	6	0	1	8	3.0	75.0
	M	0	0	0	0	0	0	0	0	0	0	0.0	0
	Unknown	2	0	2	0	1	2	0	0	0	7	2.6	NA
Total		98	43	24	26	55	16	7	0	2	271	100	70.1

The cognitive function score, routinely checked by a nurse, was derived from the medical charts for Assessment 1. In Assessment 2, the cognitive function score was checked independently within 72 h after admission, without knowing the results of Assessment 1, by a medical social worker or a medical clerk, i.e. a person of medical profession other than a nurse

### Criterion-related validity of MHLW bedriddenness ranks and cognitive function scores

**Fig. 2:**
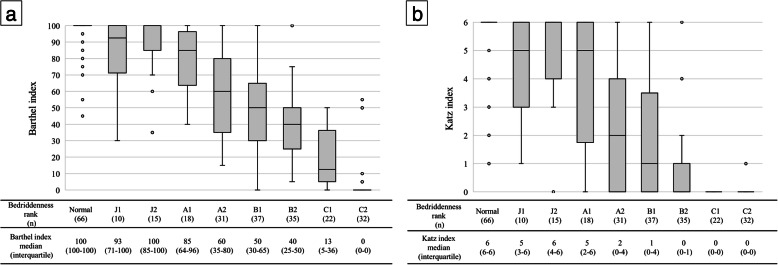
Box and Whisker diagrams showing the relationship between the MHLW bedriddenness ranks and (**a**) the BI, and **(b)** the KI. Spearman’s rank correlation coefficient between the MHLW bedriddenness ranks and the BI was −0.848, *p* < 0.001 (**a**), and between the bedriddenness ranks and the KI this was − 0.820, *p* < 0.001 (**b**)

**Fig. 3:**
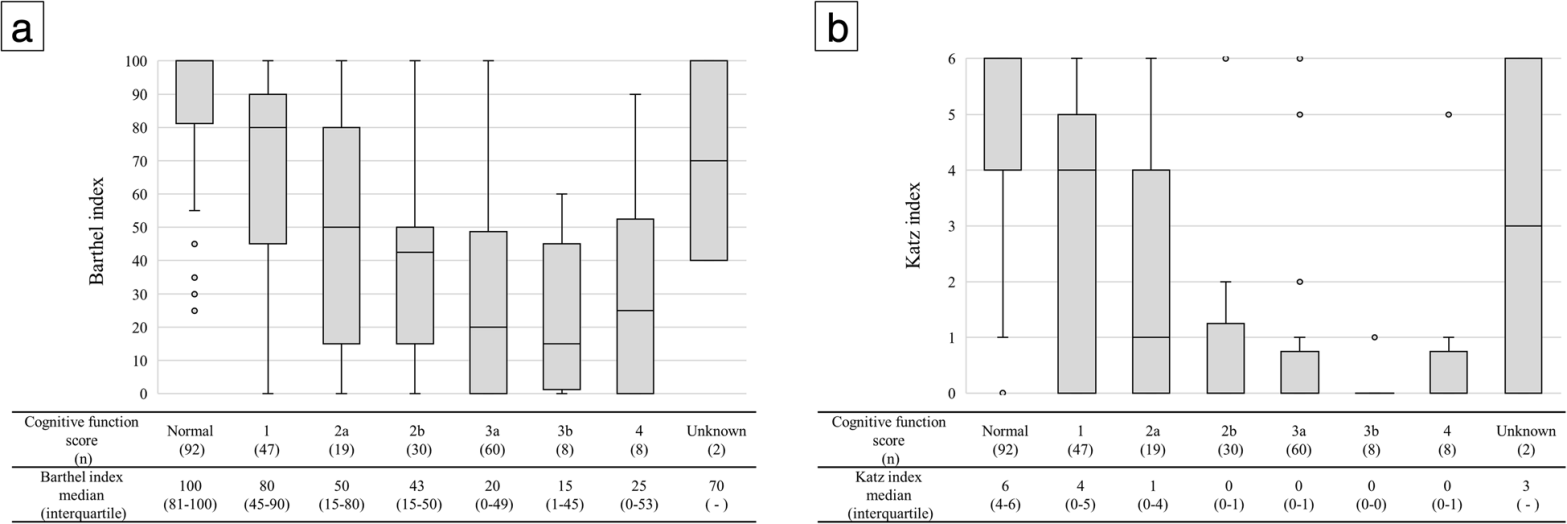
Box and Whisker diagrams showing the relationship between the MHLW cognitive function scores and (**a**) the BI, and (**b**) the KI. Spearman’s rank correlation coefficient between the MHLW cognitive function scores and the BI was − 0.667, *p* < 0.001 (**a**), and between the cognitive function scores and the KI this was − 0.661, *p* < 0.001 (**b**). No patients had a cognitive function score category of M

Bedriddenness ranks were significantly correlated with BI (r = − 0.848, *p* < 0.001; Fig. 2a) and with KI (r = − 0.820, *p* < 0.001; Fig. 2b). Cognitive function scores were significantly correlated with BI (r = − 0.667, *p* < 0.001; Fig. 3a) and with KI (r = − 0.661, *p* < 0.001; Fig. 3b).

## Discussion

A good inter-rater reliability of the MHLW bedriddenness ranks was found in this study. The BI and KI, which are well established scales to assess ADLs, have been reported to have good inter-rater reliabilities, as indicated by a Pearson’s correlation coefficient of 0.9 and by an intraclass correlation coefficient of 1.00 [7]. However, the BI takes a long time to implement due to its complexity [10], and the KI is unreliable when examining patients who require assistance for almost all of their basic ADLs [15], both of which are significant disadvantages. In comparison, the simplicity and high reliability of the MHLW bedriddenness ranks could means that this is a better scale to assess ADLs in contexts such as nursing homes or mass screenings, where many elderly patients require care for daily life, or busy clinical settings such as acute care hospitals. Additionally, bedriddenness ranks could provide us with more detailed information on patients who are bedridden by dividing them into two categories, B (Chair-bound) and C (Bed-bound) [16, 17]. However, we should note that there were relatively lower concordance rates of patients belonging to bedriddenness ranks J1, J2, and A1 between Assessment 1 and 2 than those of patients belonging to other categories. In Assessment 1, which was performed by attending nurses, information was also available from bedside observation of patients or from patients’ family members; however, in Assessment 2, which was performed by a medical social worker or medical clerk, the source of information was limited to a brief interview with patients. This highlights the need to gain information not only from patients themselves when using these easy-to-use, reliable scales, but also other sources such as from their family members, especially for patients whose conditions fluctuate according to the day and time of day.

The MHLW cognitive function scores also had an excellent inter-rater reliability, MHLW as indicated by a kappa coefficient of 0.62, Cronbach’s α of 0.87, and intraclass correlation coefficient 0.78. The MMSE [18] and ABC Dementia Scale [19] are existing measures of cognitive function that have been reported to have good inter-rater reliabilities, as shown by a kappa coefficient of 0.98 and weighted kappa coefficient of 0.75, respectively [7]. However, the MMSE consists of 11 items, including drawing pictures [7], and the ABC Dementia Scale of 13 items [19], which makes these measures cumbersome to use in extremely busy clinical settings in Japan, such as acute care hospitals or for mass screenings [5]. In contrast, cognitive function scores are simpler and easier-to-use, while also having sufficient reliability for use in mass screening or busy clinical settings, despite their slightly lower inter-rater reliability than the MMSE and ABC Dementia Scale. However, we should also be aware of the relatively lower concordance rates of patients belonging to cognitive function score categories of 2a and 2b between Assessment 1 and 2 than those of patients belonging to other categories, similarly to the case of bedriddenness ranks. Making judgements about whether patients are independent (a cognitive function score 1 or less) or require nursing care (a cognitive function score 3 or more) is straightforward due to the apparentness and unambiguousness of these scores. However, making judgements about patients with a score of 2, who could be somewhat independent under someone’s careful observation, despite certain cognitive impairments or difficulties in communication, could rely on subjective assessments, making such judgments less decisive. This characteristic of a cognitive function score of 2 meant that we relied on information gained not only from an interview with patients themselves, but also from interviews with their family members or bedside observations, to better ensure that attending nurses of Assessment 1 made the correct judgment. This also suggests that we should source information not only from patients themselves, but also from their family members or careful bedside observations, for both the cognitive function scores and bedriddenness ranks.

The MHLW bedriddenness ranks can be easier-to-use alternatives to the KI and BI. Furthermore, bedriddenness ranks were correlated with the BI and KI, which suggests that the various outcomes related to the BI and KI may also be related to the bedriddenness ranks [20–22]. Additionally, low bedriddenness ranks were significantly related to many variables in the present study and pressure ulcers, decreased ADL, malnutrition [3], oral mucosal epithelial detachment [4], and fall-related injuries in the previous studies [5]. We found that bedriddenness ranks, which generally provide a more approximate classification of ADLs than the BI or KI, were correlated with the BI and KI. However, for patients who were almost independent, with a BI of ≥80 or a KI of ≥5, the bedriddenness ranks offered a more detailed assessment of ADLs than did the BI or KI by the finer classifications of normal, J1, J2, and A1 (normal to house-bound) (Fig. 2). In a similar way, in completely bedridden patients with a KI of 0, the bedriddenness ranks gave a more detailed assessment by the finer classifications of B2 to C2.

The MHLW cognitive function scores were also significantly correlated with the BI and KI, and were related to age, sex, and location of livelihood. We did not compare cognitive function scores with other established scales of cognition, such as the MMSE, because not all inpatients completed the assessment of those established scales in this study. Thus, cognitive function scores should be compared with the standard scales of cognitive function in future research.

### Limitations

In the present study, we excluded 2906 patients due to a lack of informed consent after failing to explain the research protocol to patients themselves or their family members within 72 h after admission. This was partly due to a shortage of human resources, as well as patients’ refusal to provide informed consent. However, the characteristics of included subjects were similar to those of the overall inpatient group (S4, Table), and so we believe that this exclusion had negligible effects on the present results. The MHLW bedriddenness ranks and cognition function scores found in this study were different to those of elderly people living in care facilities [23] and those living in the local community [24]; this could be a result of selection bias, which could have resulted in an inter-rater reliability that differed from other groups of patients with different background characteristics. Furthermore, the concordance rates within each grade of bedriddenness ranks and cognitive function scores varied between Assessments 1 and 2, with low concordance rates, albeit only slightly.

## Conclusions

The MHLW bedriddenness ranks and cognitive function scores are official Japanese classifications of ADLs. Our results indicate that the implementation of these ADL grading scales are more practical than existing ones, especially in busy clinical practice or large-scale screening. Furthermore, they had a high inter-rater reliability, even among assessors with different professions, and their grades were significantly correlated with those of well-established scales to assess ADLs.

## Supplementary Information

**Additional file 1: S1, Figure.** The flowchart used by the assessor for MHLW bedriddenness ranks (A) and cognitive function scores (B).

**Additional file 2: S2, Appendix.** Characteristics of the Yuai-kai Foundation and Oda Hospital.

**Additional file 3: S3, Table.** Concordance rates between bedriddenness ranks or cognitive function scores of Assessments 1 and 2 according to the major categories of the International Classification of Diseases, 10th edition.

**Additional file 4: S4, Table.** Characteristics of all inpatients during the study period.

## Data Availability

The datasets generated and analyzed in the current study are available in the UMIN-ICDR repository, https://upload.umin.ac.jp/cgi-open-bin/icdr_e/ctr_view.cgi?recptno=R000046877

## Abbreviations

MHLW: Ministry of Health, Labour and Welfare
ADL: Activity of daily living
BI: Barthel Index
KI: Katz Index
MMSE: Mini-Mental State Examination
CI: Confidence interval
