# Supplementary material for: High inter-rater reliability of Japanese bedriddenness ranks and cognitive function scores: a hospital-based prospective observational study
Source: BMC Geriatr. 2021 Mar 9;21:168. doi: 10.1186/s12877-021-02108-x (PMC7941919; doi:10.1186/s12877-021-02108-x)
Supplement: Supplementary file 1 — Additional file 1: S1, Figure. The flowchart used by the assessor for MHLW bedriddenness ranks (A) and cognitive function scores (B). [file 12877_2021_2108_MOESM1_ESM.pptx]

## Slide 1
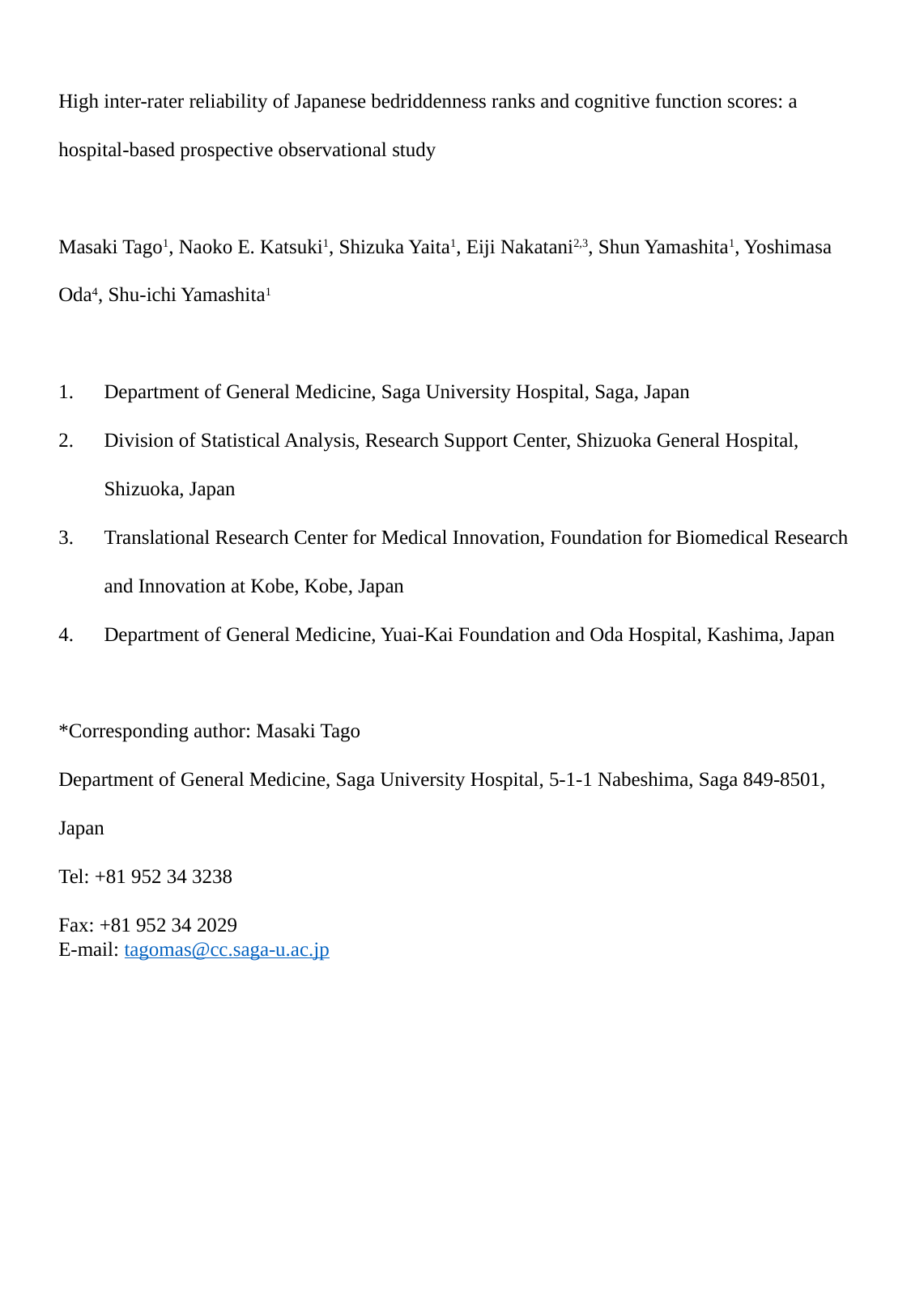

High inter-rater reliability of Japanese bedriddenness ranks and cognitive function scores: a hospital-based prospective observational study
Masaki Tago1, Naoko E. Katsuki1, Shizuka Yaita1, Eiji Nakatani2,3, Shun Yamashita1, Yoshimasa Oda4, Shu-ichi Yamashita1
Department of General Medicine, Saga University Hospital, Saga, Japan
Division of Statistical Analysis, Research Support Center, Shizuoka General Hospital, Shizuoka, Japan
Translational Research Center for Medical Innovation, Foundation for Biomedical Research and Innovation at Kobe, Kobe, Japan
Department of General Medicine, Yuai-Kai Foundation and Oda Hospital, Kashima, Japan
*Corresponding author: Masaki Tago
Department of General Medicine, Saga University Hospital, 5-1-1 Nabeshima, Saga 849-8501, Japan
Tel: +81 952 34 3238
Fax: +81 952 34 2029
E-mail: tagomas@cc.saga-u.ac.jp

## Slide 2
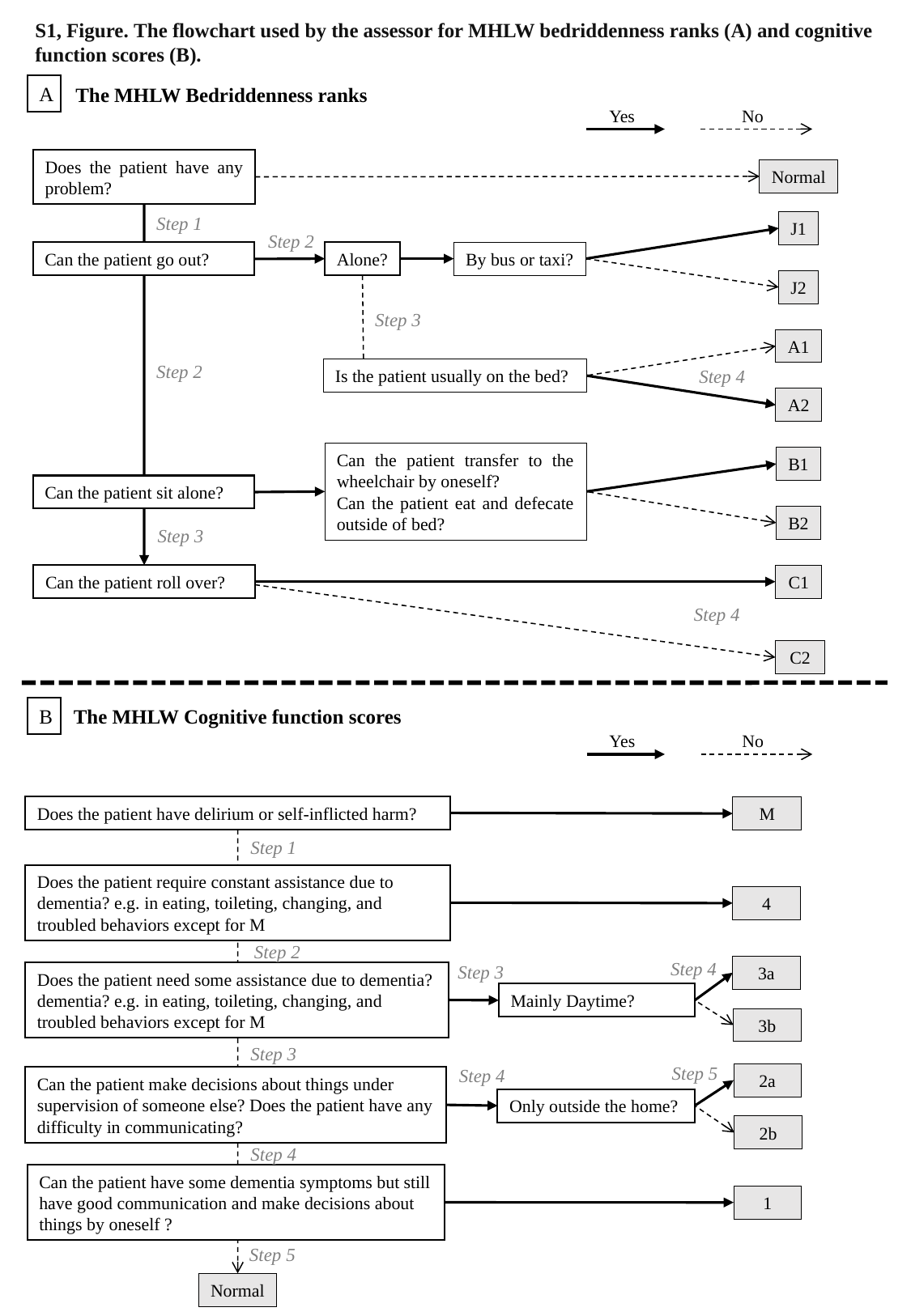

S1, Figure. The flowchart used by the assessor for MHLW bedriddenness ranks (A) and cognitive function scores (B).
A
The MHLW Bedriddenness ranks
Yes
No
Does the patient have any problem?
Normal
Step 1
J1
Step 2
Alone?
By bus or taxi?
Can the patient go out?
J2
Step 3
A1
Step 2
Step 4
Is the patient usually on the bed?
A2
Can the patient transfer to the wheelchair by oneself?
Can the patient eat and defecate outside of bed?
B1
Can the patient sit alone?
B2
Step 3
Can the patient roll over?
C1
Step 4
C2
The MHLW Cognitive function scores
B
Yes
No
Does the patient have delirium or self-inflicted harm?
M
Step 1
Does the patient require constant assistance due to dementia? e.g. in eating, toileting, changing, and troubled behaviors except for M
4
Step 2
Step 4
Step 3
3a
Does the patient need some assistance due to dementia? dementia? e.g. in eating, toileting, changing, and troubled behaviors except for M
Mainly Daytime?
3b
Step 3
Step 5
Step 4
2a
Can the patient make decisions about things under supervision of someone else? Does the patient have any difficulty in communicating?
Only outside the home?
2b
Step 4
Can the patient have some dementia symptoms but still have good communication and make decisions about things by oneself ?
1
Step 5
Normal
