# Supplementary material for: High inter-rater reliability of Japanese bedriddenness ranks and cognitive function scores: a hospital-based prospective observational study
Source: BMC Geriatr. 2021 Mar 9;21:168. doi: 10.1186/s12877-021-02108-x (PMC7941919; doi:10.1186/s12877-021-02108-x)
Supplement: Supplementary file 2 — Additional file 2: S2, Appendix. Characteristics of the Yuai-kai Foundation and Oda Hospital. [file 12877_2021_2108_MOESM2_ESM.docx]

High inter-rater reliability of Japanese bedriddenness ranks and cognitive function scores: a hospital-based prospective observational study

Masaki Tago^1^, Naoko E. Katsuki^1^, Shizuka Yaita^1^, Eiji Nakatani^2,3^, Shun Yamashita^1^, Yoshimasa Oda^4^, Shu-ichi Yamashita^1^

1. Department of General Medicine, Saga University Hospital, Saga, Japan
2. Division of Statistical Analysis, Research Support Center, Shizuoka General Hospital, Shizuoka, Japan
3. Translational Research Center for Medical Innovation, Foundation for Biomedical Research and Innovation at Kobe, Kobe, Japan
4. Department of General Medicine, Yuai-Kai Foundation and Oda Hospital, Kashima, Japan

*Corresponding author: Masaki Tago

Department of General Medicine, Saga University Hospital, 5-1-1 Nabeshima, Saga 849-8501, Japan

Tel: +81 952 34 3238

Fax: +81 952 34 2029

E-mail: [tagomas@cc.saga-u.ac.jp](mailto:tagomas@cc.saga-u.ac.jp)

**S2, Appendix. Characteristics of the Yuai-kai Foundation and Oda Hospital.**

The hospital comprises 10 departments with 111 beds, including the Departments of Internal Medicine, General Surgery, and Cardiovascular Surgery, but not the Department of Orthopedic Surgery. The hospital is in a suburban city, Kashima, in Saga prefecture in southern Japan, which has an approximate population of 90,000, and annually treats over 3,100 inpatients.
