# Supplementary material for: High inter-rater reliability of Japanese bedriddenness ranks and cognitive function scores: a hospital-based prospective observational study
Source: BMC Geriatr. 2021 Mar 9;21:168. doi: 10.1186/s12877-021-02108-x (PMC7941919; doi:10.1186/s12877-021-02108-x)
Supplement: Supplementary file 3 — Additional file 3: S3, Table. Concordance rates between bedriddenness ranks or cognitive function scores of Assessments 1 and 2 according to the major categories of the International Classification of Diseases, 10th edition. [file 12877_2021_2108_MOESM3_ESM.docx]

High inter-rater reliability of Japanese bedriddenness ranks and cognitive function scores: a hospital-based prospective observational study

Masaki Tago^1^, Naoko E. Katsuki^1^, Shizuka Yaita^1^, Eiji Nakatani^2,3^, Shun Yamashita^1^, Yoshimasa Oda^4^, Shu-ichi Yamashita^1^

1. Department of General Medicine, Saga University Hospital, Saga, Japan
2. Division of Statistical Analysis, Research Support Center, Shizuoka General Hospital, Shizuoka, Japan
3. Translational Research Center for Medical Innovation, Foundation for Biomedical Research and Innovation at Kobe, Kobe, Japan
4. Department of General Medicine, Yuai-Kai Foundation and Oda Hospital, Kashima, Japan

*Corresponding author: Masaki Tago

Department of General Medicine, Saga University Hospital, 5-1-1 Nabeshima, Saga 849-8501, Japan

Tel: +81 952 34 3238

Fax: +81 952 34 2029

E-mail: [tagomas@cc.saga-u.ac.jp](mailto:tagomas@cc.saga-u.ac.jp)

**S3, Table. Concordance rates between bedriddenness ranks or cognitive function scores of Assessments 1 and 2 according to the major categories of the International Classification of Diseases, 10th edition.**

| the major categories of ICD-10 | Number | Concordance rate  of bedrriddenness rank | Concordance rate  of cognitive function score |
| --- | --- | --- | --- |
| Certain Infectious and Parasitic Diseases | 12 | 83.3% | 75.0% |
| Neoplasms | 25 | 68.0% | 84.0% |
| Diseases of the blood and blood-forming organs and certain disorders involving the immune mechanism | 6 | 66.7% | 33.3% |
| Endocrine, nutritional and metabolic diseases | 19 | 66.7% | 63.2% |
| Mental, Behavioral and Neurodevelopmental disorders | 1 | 0.0% | 100% |
| Diseases of the nervous system | 10 | 66.7% | 80.0% |
| Diseases of the ear and mastoid process | 4 | 100% | 75.0% |
| Diseases of the circulatory system | 63 | 65.1% | 73.0% |
| Diseases of the respiratory system | 57 | 70.9% | 68.4% |
| Diseases of the digestive system | 23 | 65.2% | 73.9% |
| Diseases of the skin and subcutaneous tissue | 12 | 63.6% | 50.0% |
| Diseases of the musculoskeletal system and connective tissue | 3 | 66.7% | 100% |
| Diseases of the genitourinary system | 15 | 80.0% | 73.3% |
| Symptoms, signs and abnormal clinical and laboratory findings, not elsewhere classified | 3 | 100% | 33.3% |
| Injury, poisoning and certain other consequences of external causes | 18 | 50.0% | 61.1% |
| Total | 271 | 68.0% | 70.1% |
| p-value^†^ |  | 0.681 | 0.415 |

†P-value was calculated by chi-square test. There was no significant difference among the concordance rates according to the major categories of ICD-10 in both bedrriddenness rank and cognitive function scores. The concordance rates were calculated between Assessment 1 and 2. ICD-10: International Classification of Diseases, 10th edition.
