# Supplementary material for: High inter-rater reliability of Japanese bedriddenness ranks and cognitive function scores: a hospital-based prospective observational study
Source: BMC Geriatr. 2021 Mar 9;21:168. doi: 10.1186/s12877-021-02108-x (PMC7941919; doi:10.1186/s12877-021-02108-x)
Supplement: Supplementary file 4 — Additional file 4: S4, Table. Characteristics of all inpatients during the study period. [file 12877_2021_2108_MOESM4_ESM.docx]

High inter-rater reliability of Japanese bedriddenness ranks and cognitive function scores: a hospital-based prospective observational study

Masaki Tago^1^, Naoko E. Katsuki^1^, Shizuka Yaita^1^, Eiji Nakatani^2,3^, Shun Yamashita^1^, Yoshimasa Oda^4^, Shu-ichi Yamashita^1^

1. Department of General Medicine, Saga University Hospital, Saga, Japan
2. Division of Statistical Analysis, Research Support Center, Shizuoka General Hospital, Shizuoka, Japan
3. Translational Research Center for Medical Innovation, Foundation for Biomedical Research and Innovation at Kobe, Kobe, Japan
4. Department of General Medicine, Yuai-Kai Foundation and Oda Hospital, Kashima, Japan

*Corresponding author: Masaki Tago

Department of General Medicine, Saga University Hospital, 5-1-1 Nabeshima, Saga 849-8501, Japan

Tel: +81 952 34 3238

Fax: +81 952 34 2029

E-mail: [tagomas@cc.saga-u.ac.jp](mailto:tagomas@cc.saga-u.ac.jp)

**S4, Table. Characteristics of all inpatients during the period.**

| Variables | n | % (interquartile) |
| --- | --- | --- |
| Age (year, median) | 85 | (80 - 90) |
| Gender, Male | 1,386 | 43.0 |
| By ambulance | 546 | 16.9 |
| Referral medical letter | 1,097 | 34.0 |
| Length of hospital stay (day, median) | 11 | (6 - 20) |
| Departments |  |  |
| Internal medicine | 1,960 | 60.8 |
| Surgery | 388 | 12.0 |
| Cardiovascular surgery | 359 | 11.1 |
| Dermatology | 169 | 5.2 |
| Plastic surgery | 167 | 5.2 |
| Otolaryngology | 82 | 2.5 |
| Neurosurgery | 96 | 3.0 |
| Bedriddenness rank (Assessment 1) |  |  |
| Normal | 1,002 | 31.1 |
| J1 | 177 | 5.5 |
| J2 | 198 | 6.1 |
| A1 | 299 | 9.3 |
| A2 | 307 | 9.5 |
| B1 | 311 | 9.7 |
| B2 | 267 | 8.3 |
| C1 | 223 | 6.9 |
| C2 | 369 | 11.5 |
| Unknown | 69 | 2.1 |
| Cognitive function score (Assessment 1) |  |  |
| Normal | 1,421 | 44.1 |
| 1 | 546 | 16.9 |
| 2a | 170 | 5.3 |
| 2b | 283 | 8.8 |
| 3a | 566 | 17.6 |
| 3b | 79 | 2.5 |
| 4 | 68 | 2.1 |
| M | 8 | 0.2 |
| Unknown | 81 | 2.5 |

Categorical variables are presented as the numbers of patients and percentages, and continuous variables are presented as the median and quartile ranges.
